# Outcomes of Inpatient Chemotherapy for Patients with Newly Diagnosed Extensive-Stage Small-Cell Lung Cancer

**DOI:** 10.3390/curroncol33070388

**Published:** 2026-06-26

**Authors:** Sara N. Gauthier, Paul Wheatley-Price, David J. Stewart, Stephanie Brule, Mikaela Ney, Garth Nicholas, Sara M. Moore

**Affiliations:** 1 Faculty of Medicine, NOSM University, 935 Ramsey Lake Rd., Sudbury, ON P3E 2C6, Canada; sargauthier@nosm.ca; 2 Division of Medical Oncology, The Ottawa Hospital, 501 Smyth Rd., Ottawa, ON K1H 8L6, Canada; pwheatleyprice@toh.ca (P.W.-P.); dstewart@toh.ca (D.J.S.); sbrule@toh.ca (S.B.); gnicholas@toh.ca (G.N.); 3 Cancer Therapeutics Program, Ottawa Hospital Research Institute, 1053 Carling Ave, Ottawa, ON K1Y 4E9, Canada

**Keywords:** small-cell lung cancer, tumor lysis syndrome, inpatient

## Abstract

**Background:** Small-cell lung cancer (SCLC) accounts for 15% of lung cancers, with 70% diagnosed at extensive-stage (ES). Systemic therapy is often considered in very unwell patients, although outcomes for inpatients with ES-SCLC are not well understood. **Methods:** We reviewed patients with de novo ES-SCLC who had an inpatient medical oncology consultation at the Ottawa Hospital between 2013 and 2021. The primary endpoint was overall survival (OS). Secondary endpoints included length of stay (LOS) and tumor lysis syndrome (TLS) incidence. **Results:** There were 127 patients identified. Median age was 68 years (range 50–87), 58% female, 99% had prior smoking history, 22% had brain metastases, and 64% had liver metastases. Ninety-two (72%) received chemotherapy. Median OS for treated patients was 5.9 months (95% CI, 4.5–7.3 m), and a median LOS of 13 days. Patients in the non-treatment cohort had a median OS of 14 days (95% CI, 0.2–0.7 m), a median LOS of 11 days, and 54% in-hospital death rate. TLS occurred in six of the 76 (8%) evaluated patients, all dying within 7 days of chemotherapy. **Conclusions:** Chemotherapy was associated with longer survival among inpatients with ES-SCLC. TLS was rare but uniformly fatal, highlighting the need for aggressive prophylaxis among patients with identified risk factors.

## Simple Summary

SCLC is an aggressive form of lung cancer, and as a result, many patients are highly symptomatic at diagnosis. In this study, we review patients with recently diagnosed SCLC who are admitted to hospital. In total, 72% of patients were treated with chemotherapy and had a median survival of 6 months. We note that 8% developed tumor lysis syndrome, which is higher than expected from other studies. All the patients with tumor lysis syndrome died within 7 days of treatment. This study highlights the treatment and outcomes of a patient population that is not commonly studied and can help clinicians counsel patients considering treatment. These results will also assist with optimal prophylaxis and management of TLS and identify TLS in SCLC as an area of need for further research.

## 1. Introduction

Lung cancer is the leading cause of cancer death in North America. Small-cell lung cancer (SCLC) accounts for 15% of all lung cancers, of which 66% of patients have extensive-stage (ES) disease at diagnosis [1]. SCLC is characterized by rapid proliferation, early metastases, and a 2-year overall survival (OS) of less than 10% among patients with ES disease [2]. Platinum–etoposide therapy has been the standard of care for treatment of ES-SCLC in the last three decades, with recent evidence showing survival benefit with the addition of immunotherapy [3,4].

SCLC is highly chemosensitive and can respond to therapy rapidly, such that treatment may be considered in patients who are hospitalized or are very unwell [1]. However, there is a paucity of data describing systemic therapy outcomes for inpatients with ES-SCLC. Landmark trials examining the use of immunotherapy for ES-SCLC, including CASPIAN and IMpower133, exclude patients with poor performance status, which may limit their generalizability to the inpatient setting [3,4,5]. Further, in our jurisdiction immunotherapy is only publicly funded in the outpatient setting.

Tumor lysis syndrome (TLS) is a condition where tumor cells release their contents into the bloodstream, most commonly following systemic therapy induction, causing increased uric acid and electrolyte abnormalities that can lead to renal insufficiency, seizures, arrythmias and death. TLS is classically associated with acute leukemias and high-grade lymphomas with less frequent reports in solid tumors. However, expert guidelines classify patients with SCLC as having intermediate baseline risk (~1–5%) of developing TLS, likely due to its highly proliferative nature, chemosensitivity, and predilection for bulky disease [6,7,8]. Other risk factors for TLS include older age, elevated lactate dehydrogenase (LDH), and pre-existing hyperuricemia, dehydration, or renal impairment [7]. The prior literature describing TLS following systemic therapy induction among patients with SCLC is limited to case reports [9,10,11,12,13,14,15,16,17]. TLS may be underrecognized in patients with SCLC, for which standardized prophylactic regimens have not been established.

This retrospective review aims to evaluate treatment patterns, TLS risk, and survival outcomes for patients with ES-SCLC diagnosed in the inpatient setting. The abstract of this work was previously presented at the World Conference for Lung Cancer in 2024 [18]. The present study provides expanded methodology, additional analyses, and complete results.

## 2. Materials and Methods

A retrospective review was conducted of all patients with pathologically confirmed de novo ES-SCLC who had an inpatient medical oncology consultation at the Ottawa Hospital (TOH) in Ottawa, Canada between January 2013 and December 2021. TOH is the sole provider of oncologic services in the region, covering a catchment area that includes over 1.1 million people. Data on baseline demographics, staging, treatment information, and outcomes were retrieved from local contributions to the Canadian Small-Cell Lung Cancer Database (CASCADE) [19]. Further variables were collected by manual review of electronic medical records. Clinical notes, laboratory testing, and imaging investigations were available through the electronic medical record for all patients. Medication dosing information was available from June 2019 onward, and inconsistently available before then. Patients with uncertain primary sites or those diagnosed at other institutions were excluded.

The primary endpoint was overall survival among patients receiving systemic therapy versus those who did not. Secondary endpoints included hospital length of stay (LOS), discharge disposition, and the incidence of tumor lysis syndrome defined by the Cairo–Bishop criteria [19]. Patients were considered evaluable for TLS if they had 3 or more of the following laboratory values within 7 days of systemic therapy initiation: calcium, potassium, phosphate, and uric acid. Clinical response was derived from radiology reports and clinician documentation, with clinical benefit indicating stable disease, a partial response, or complete response as per the treating physician. Clinic visits and imaging investigations were scheduled at the discretion of the treating physician.

Statistical analysis was performed using SPSS version 29 and the Kaplan–Meier Estimator from StatsCalculators [20]. Descriptive statistics were used to summarize baseline demographics, staging, and treatment. Baseline prognostic factors were compared between cohorts using the Chi-Square or Fisher’s exact test for categorical variables and the Mann–Whitney test for continuous variables. Overall survival was estimated using the Kaplan–Meier method, and survival curves compared with a log-rank test. A sensitivity analysis was performed for overall survival restricted to patients with poor performance status (ECOG 3–4) and baseline liver metastases. Patients were censored as of the last date they were known to be alive. Landmark survival proportions at 1, 3, and 6 months were estimated. Ethics approval was obtained from the Ottawa Hospital Research Institute Research Ethics Board.

## 3. Results

### 3.1. Patient Characteristics 

During the study period, 127 patients were identified. Baseline demographics and staging information are provided in Table 1. Most patients had current or former smoking history with TNM stage IV disease, with similar age and sex distribution between cohorts. Patients not receiving systemic therapy had significantly poorer Eastern Cooperative Oncology Group performance status (ECOG PS) compared to those that received systemic therapy (*p* < 0.001), with 30 (88%) and 47 (53%) patients with an ECOG PS of 3–4, respectively.

### 3.2. Treatment Patterns

Treatment patterns are summarized in Table 2. Overall, 92 (72%) received chemotherapy and 35 (28%) did not. Of the 92 patients who received first-line treatment, most completed one (n = 78, 85%) or two (n = 11, 12%) inpatient chemotherapy cycles. The predominant first-line regimen was etoposide with carboplatin (n = 52, 57%) or cisplatin (n = 35, 38%). Among the patients with available dosing information (n = 31), 42% received a dose reduction of platinum and/or etoposide. Approximately two-thirds of the patients receiving systemic therapy experienced clinical benefit. Eighteen (20%) patients subsequently received a second line of systemic therapy.

Of the 35 patients who did not receive systemic therapy, eight (23%) were offered chemotherapy but decided against it, two (6%) were admitted for symptomatic brain metastasis and systemic therapy was not urgently required, one (3%) had unknown reason, and the remaining 25 (71%) were not offered chemotherapy due to a combination of poor functional status, comorbidities, and rapid clinical deterioration.

### 3.3. Disposition

The median length of hospitalization was similar between the cohorts: 14 days (range 4–84) for the treated patients and 11 days (1–30) for those not treated. More than half of the non-treatment cohort died during their admission (n = 19, 54%), compared to 20% of those in the systemic therapy cohort (n = 18). A greater proportion of treated patients (n = 57, 62%) were discharged home compared to those not receiving systemic therapy (n = 6, 17%). Other discharge dispositions in the treatment and non-treatment cohorts included hospice (1% vs. 17%), rehabilitation facility (9% vs. 0%), regional hospital (3% vs. 6%), retirement home (5% vs. 3%), and unknown (0% vs. 3%).

### 3.4. Tumor Lysis Syndrome

Of the 92 patients that received systemic therapy, 76 patients (83%) had available labs permitting evaluation for TLS. Of these, six patients developed TLS (8%), all of whom died within seven days of systemic therapy initiation. All had liver metastases and baseline LDH > 1500 IU/L (unknown for one patient). TLS developed in 6/43 (14%) patients with liver metastases, and 5/8 (63%) of patients with baseline LDH ≥ 1000 IU/L. Of note, 53% (n = 49) of patients receiving systemic therapy had an unknown LDH at the time of diagnosis. 

Among patients with unknown LDH at diagnosis (n = 49), 41 patients (84%) were evaluable for TLS, of which one patient (2.4%) developed TLS. There were 43 patients with baseline LDH. Thirty-five (82%) were evaluable for TLS, of which five (14%) developed TLS.

For the 25 patients enrolled when detailed medication records were available, 60% (n = 15) received prophylactic allopurinol. Of the six patients who developed TLS, all received prophylactic allopurinol and one also received prophylactic rasburicase. To manage TLS, five received intravenous fluids, three received rasburicase, and one patient from each group required dialysis and ICU transfer following the onset of TLS.

### 3.5. Survival Outcomes

**Figure 1:**
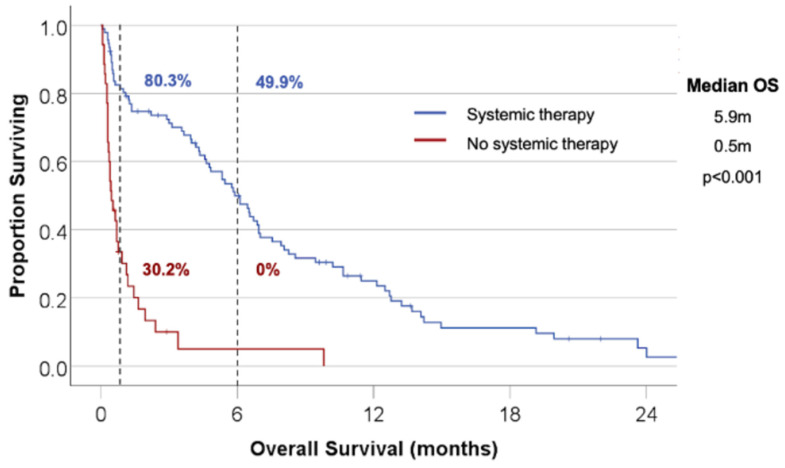
Kaplan–Meier curve depicting overall survival (OS) at 30 days and 6 months among patients who underwent systemic therapy versus those who did not.

The Kaplan–Meier curve depicting median OS is provided in Figure 1. Overall median OS was 4.0 months (m) (95% CI, 2.5–5.5 m). The systemic therapy cohort had a median OS of 5.9 m (95% CI, 4.5–7.3 m, *p* < 0.001), 1 m OS of 80%, 3 m OS of 71%, and 6 m OS of 50%. In contrast, the patients not receiving systemic therapy had a median OS of 14 days (95% CI, 0.2–0.7 m, *p* < 0.001), 1 m OS of 30%, and 3 m OS of 10%. All the patients not receiving systemic therapy died within 6 months of diagnosis.

A sensitivity analysis was performed evaluating overall survival restricted to patients with poor performance status (ECOG 3–4) and baseline liver metastases (Supplementary Figure S1). Median OS was 1.1 months (m) (95% CI, 0.6–5.3 m) in the overall population. The systemic therapy cohort had a median OS of 5.5 m (95% CI, 3.6–11.5, *p* < 0.001) compared to 12 days (95% CI, 0.3–0.7 m, *p* < 0.001) in the patients not receiving systemic therapy.

## 4. Discussion

Our findings demonstrate that 72% of inpatients with ES-SCLC underwent chemotherapy with an associated median OS of 5.9 months. The patients not receiving systemic therapy had significantly poorer ECOG PS, a median OS of 14 days, and a death rate of over 50% while admitted to hospital.

Unsurprisingly, 90% of our hospitalized study population had an ECOG PS of ≥2, compared to 35% of patients with ES-SCLC reported in a real-world cohort including patients in both inpatient and outpatient settings [21]. The landmark trials IMpower133 and CASPIAN, which cemented chemoimmunotherapy as the new standard of care for extensive-stage SCLC, excluded patients with an ECOG of ≥2 [3,4]. There have been a number of retrospective studies examining the use of chemoimmunotherapy for patients with ECOG ≥ 2 [22,23,24]. These studies show that patients with ECOG ≥ 2 still benefit from the addition of immunotherapy, though there are conflicting results about whether ECOG ≥ 2 is an independent predictor of reduced survival outcomes. It is notable that most patients in these studies were ECOG 2, with only small numbers of patients with ECOG 3–4. This is in contrast to our hospitalized population, which included 61% of patients with ECOG 3 or higher. Our study was done prior to the widespread availability of immunotherapy for ES-SCLC, so only two patients had immunotherapy incorporated into their first-line treatment. Immunotherapy is not funded at our center for patients admitted to the hospital, but can be added if the patients improve and are discharged from the hospital. It is unclear if the addition of immunotherapy will significantly impact survival outcomes in this subpopulation with very poor performance status and will be an area of focus for future work.

The observed survival for the treated patients was similar to a previous study evaluating patients with ES-SCLC receiving inpatient systemic therapy, reporting a median OS of 5.7 months (3.7–6.9 m) [25]. In comparison, a real-world study of patients with ES-SCLC reported a median OS of 7.9 m and 8.2 m for those receiving etoposide with carboplatin or cisplatin, respectively [2]. The median OS of the control arm in both the CASPIAN and IMpower133 clinical trials was 10.3 m [3,4]. The poorer survival among inpatients compared to population-based studies or clinical trials likely relates to the higher presence of poor prognostic factors such as impaired ECOG performance status and increased frequency of liver metastasis. The survival for patients not receiving systemic therapy was very poor, which may be a combination of the aggressive biology of untreated SCLC and appropriate physician selection of patients with guarded prognosis or lack of fitness to endure systemic therapy.

The treatment landscape for SCLC has changed in recent years, with both the introduction of tarlatamab, a bispecific T-cell engager, and evidence to support maintenance lurbinectedin. In the DeLLphi-304 study, treatment with tarlamatab significantly improved overall survival compared to standard chemotherapy for patients whose disease had progressed after initial platinum-based chemotherapy [26]. The IMforte trial showed improvements in overall survival when lurbinectedin was added to atezolizumab for patients treated with initial chemotherapy plus atezolizumab [27]. These treatments are not yet publicly reimbursed in Canada but once approved will likely shift the overall population-based survival outcomes for ES-SCLC. Among the patients who were treated with initial chemotherapy in our study, 20% went on to receive a second line of therapy, which is similar to prior population-based Canadian studies [28]. Although these patients are unwell at diagnosis, many improve with treatment and may become eligible for these newer therapies. This is the first retrospective review to assess the incidence of TLS in this patient population, highlighting a concerning mortality rate among affected patients. In the last 40 years, the previous literature describing patients with SCLC who develop TLS after systemic therapy induction is limited to case reports [9,10,11,12,13,14,15,16,17]. The scarce data regarding TLS in patients with SCLC may result in inadequate prophylaxis or delays in its recognition and treatment, the effects of which can be devastating.

While patients with SCLC are generally classified as having intermediate risk (~1–5%) of developing TLS at baseline, individual risk assessments are essential to guide appropriate prophylaxis [7,8]. Escalation to a high-risk category (TLS risk > 5%) is recommended for patients with renal insufficiency or baseline elevations in uric acid, potassium, or phosphate [8]. In our cohort, TLS occurred in 8% of the evaluated patients following chemotherapy initiation, resulting in death for all the affected patients. These patients consistently presented with liver metastases, poor ECOG PS, and markedly elevated LDH (unknown for 1). Given these findings, we recommend that clinicians should be diligent in undertaking full risk stratification for patients with a high burden of liver metastases or baseline serum LDH > 3× ULN, as they may be high-risk for TLS.

A 2023 consensus guideline by Perissinotti et al. outlines recommendations for prophylaxis and management of TLS. For inpatients, TLS monitoring laboratory investigations are recommended every 8–12, 6 or 4 h for patients with intermediate risk, high risk, or for those with established TLS, respectively [7]. Standard TLS prophylaxis was not routinely implemented in our study population. Continuous IV fluids (2–3 L/m^2^ daily) are recommended for all inpatients with SCLC prior to systemic therapy initiation [7]. For patients with intermediate risk, allopurinol should be initiated 2–3 days prior to systemic therapy [7]. If more urgent systemic therapy is required, rasburicase could be used instead of allopurinol. Notably, all the patients with established TLS in our study had >72 h between diagnosis and systemic therapy initiation, allowing time for the initiation of allopurinol.

Rasburicase prophylaxis should be considered in patients at high risk of developing TLS who have baseline hyperuricemia, although it is contraindicated in patients with G6PD deficiency [7]. Rasburicase prophylaxis was only given to one patient in our study, and is only approved by our hospital for patients with elevated uric acid or established TLS. It is unclear if wider use of rasburicase would have changed the outcome for any patients in our study.

The cost differential of TLS prophylaxis with rasburicase compared to allopurinol is a potential area of concern when considering more widespread use of rasburicase. However, the clinical and economic consequences of TLS are considerable, underscored by the 100% mortality rate observed in our study population. Rasburicase has demonstrated superior efficacy in lowering serum uric acid levels, coupled with a significantly faster onset of action compared to allopurinol [29]. Most existing cost analyses focus on TLS management rather than prophylaxis. One retrospective cohort found that patients with established clinical or laboratory TLS treated with rasburicase monotherapy incurred significantly lower hospitalization costs—averaging a $20,038 reduction—compared to those treated with allopurinol [29]. This study also found significantly shorter intensive care and total hospital stays among rasburicase-treated patients [29]. The superior urate-lowering effects and devastating outcome of established TLS in our population may lend support for the use of rasburicase for TLS prophylaxis in high-risk patients with SCLC. Further prospective studies are needed to optimize prophylactic strategies for TLS in SCLC.

Our study is limited by its retrospective design. There may be unrecognized confounders contributing to the differences observed between cohorts. Evaluating the incidence, prophylaxis, and treatment of TLS was limited to patients diagnosed after the introduction of a new electronic medical records system at our institution in June 2019. Many patients did not have a baseline LDH available, limiting our ability to perform a robust risk factor analysis. Further, only two patients received immunotherapy in addition to platinum–etoposide, as the study period preceded widespread public access to immunotherapy at our institution. Because of poor PS, the majority of these patients would not have been eligible for pivotal immunotherapy studies. Future work evaluating outcomes for hospitalized patients treated with modern immunotherapy-containing regimens will be important. The strengths of this study include a large sample size, a diverse population, and the identification of six patients with ES-SCLC who developed TLS following systemic therapy. Larger studies are needed to further evaluate predictive factors, prophylaxis, and the treatment of TLS in patients with ES-SCLC.

In conclusion, chemotherapy was associated with longer survival among inpatients with ES-SCLC. TLS was rare but uniformly fatal, highlighting the need for aggressive prophylaxis among patients with identified risk factors.

## Figures and Tables

**Table 1:** Baseline demographics and staging information.

Variable	Overall (n = 127)	Systemic Therapy (n = 92)	No Systemic Therapy (n = 35)	*p*-Value
Age, median (range)	68 (50–87)	68 (50–86)	70 (53–87)	0.651
Sex Male Female	53 (42) 74 (58)	38 (41) 54 (59)	15 (43) 20 (57)	0.874
Smoking status Never Former Current	1 (1) 54 (44) 67 (55)	0 38 (43) 51 (57)	1 (3) 16 (49) 16 (49)	0.219
Year of diagnosis 2013–2017 2018–2021	74 (58) 53 (42)	53 (58) 39 (42)	21 (60) 14 (40)	0.807
TNM ^1^ Stage III IV	4 (3) 123 (97)	4 (4) 88 (96)	0 35 (100)	0.575
Brain metastases	27 (22)	17 (19)	10 (31)	0.140
Liver metastases	80 (64)	56 (62)	24 (69)	0.463
ECOG PS ^2^ 0–1 2 3 4	12 (10) 35 (28) 51 (41) 26 (21)	10 (11) 33 (37) 34 (39) 13 (14)	2 (6) 2 (6) 17 (50) 13 (38)	<0.001
Creatinine umol/L, median (range)	69 (27–709)	68.5 (27–560)	77 (27–709)	0.413
Bilirubin umol/L, median (range)	8 (2–192)	8 (2–135)	13 (4–192)	0.010

^1^ TNM: tumor node metastasis; ^2^ ECOG PS: Eastern Cooperative Oncology Group performance status.

**Table 2:** Summary of treatment patterns among patients who underwent systemic therapy (n = 92).

Variable		Systemic Therapy (n = 92)
Thoracic radiation	Consolidative	21 (23)
	Palliative	11 (12)
	None	58 (63)
	Unknown	2 (2)
Prophylactic cranial irradiation	Yes	14 (15)
	No	76 (83)
	Unknown	2 (2)
First-line chemotherapy regimen	Cisplatin/etoposide	35 (38)
	Carboplatin/etoposide ^3^	52 (57)
	Platinum NOS/etoposide	1 (1)
	Carboplatin/irinotecan	2 (2)
	CAV	2 (2)
First line: number of cycles	1	26 (28)
	2	12 (13)
	3	7 (8)
	4+	47 (51)
First line: number of inpatient cycles	0 ^4^	2 (2)
	1	78 (85)
	2	11 (12)
	3	1 (1)
	4+	0
First line: clinical response	Clinical benefit	59 (64)
	No clinical benefit	8 (9)
	Death	10 (11)
	Unknown	13 (14)
Second-line systemic therapy	Yes	18 (20)
	No	70 (76)
	Unknown	4 (4)

^3^ Two patients had immunotherapy (atezolizumab, durvalumab) added later in the treatment course; ^4^ two patients had a medical oncology consultation in the inpatient setting and received systemic therapy shortly after discharge; CAV: cyclophosphamide, adriamycin, vincristine; NOS: not otherwise specified.

## Data Availability

The data presented in this study are available on request from the corresponding author. Data are not publicly available due to privacy restrictions.

## Supplementary Materials

The following supporting information can be downloaded at https://www.mdpi.com/article/10.3390/curroncol33070388/s1, Figure S1: Kaplan-Meier curve depicting overall survival among patients who underwent systemic therapy versus those that did not. Restricted to patients with ECOG PS 3–4 and baseline liver metastases.

## Abbreviations

The following abbreviations are used in this manuscript:

CAV	Cyclophosphamide, adriamycin, vincristine
ECOG PS	Eastern Cooperative Oncology Group performance status
ES	Extensive stage
OS	Overall survival
SCLC	Small-cell lung cancer
TLS	Tumor lysis syndrome
