# Supplementary figures and images for: Outcomes of Inpatient Chemotherapy for Patients with Newly Diagnosed Extensive-Stage Small-Cell Lung Cancer
Source: Curr Oncol. 2026 Jun 26;33(7):388. doi: 10.3390/curroncol33070388 (PMC13409700; doi:10.3390/curroncol33070388)

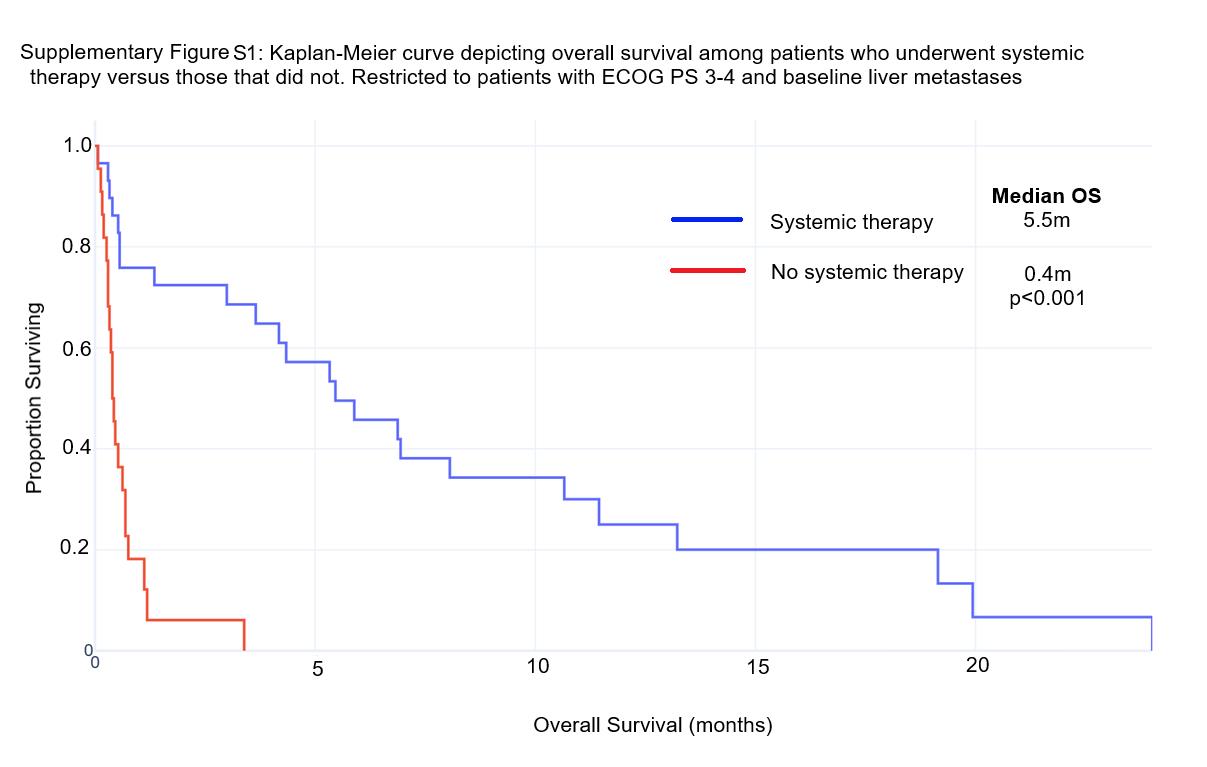

Supplement: Supplementary file 1 [file curroncol-33-00388-s001.zip › curroncol-4350246-supplementary.jpg]
